# Accuracy and Bias in Artificial Intelligence Chatbot Recommendations for Oculoplastic Surgeons

**DOI:** 10.7759/cureus.57611

**Published:** 2024-04-04

**Authors:** Alomi O Parikh, Michael C Oca, Jordan R Conger, Allison McCoy, Jessica Chang, Sandy Zhang-Nunes

**Affiliations:** 1 Ophthalmology, USC Roski Eye Institute, Keck School of Medicine, University of Southern California, Los Angeles, USA; 2 Ophthalmology, University of California San Diego School of Medicine, La Jolla, USA; 3 Oculofacial Plastic Surgery, USC Roski Eye Institute, Keck School of Medicine, University of Southern California, Los Angeles, USA; 4 Oculofacial Plastic Surgery, Del Mar Plastic Surgery, San Diego, USA; 5 Oculofacial Plastic Surgery, USC Roski Eye Institute, Keck School Medicine, University of Southern California, Los Angeles, USA; 6 Ophthalmology, USC Roski Eye Institute, Keck School Medicine, University of Southern California, Los Angeles, USA

**Keywords:** surgeon recommendations, doctor search, doctor recommendations, chatbots, artificial intelligence

## Abstract

Purpose

The purpose of this study is to assess the accuracy of and bias in recommendations for oculoplastic surgeons from three artificial intelligence (AI) chatbot systems.

Methods

ChatGPT, Microsoft Bing Balanced, and Google Bard were asked for recommendations for oculoplastic surgeons practicing in 20 cities with the highest population in the United States. Three prompts were used: “can you help me find (an oculoplastic surgeon)/(a doctor who does eyelid lifts)/(an oculofacial plastic surgeon) in (city).”

Results

A total of 672 suggestions were made between (oculoplastic surgeon; doctor who does eyelid lifts; oculofacial plastic surgeon); 19.8% suggestions were excluded, leaving 539 suggested physicians. Of these, 64.1% were oculoplastics specialists (of which 70.1% were American Society of Ophthalmic Plastic and Reconstructive Surgery (ASOPRS) members); 16.1% were general plastic surgery trained, 9.0% were ENT trained, 8.8% were ophthalmology but not oculoplastics trained, and 1.9% were trained in another specialty. 27.7% of recommendations across all AI systems were female.

Conclusions

Among the chatbot systems tested, there were high rates of inaccuracy: up to 38% of recommended surgeons were nonexistent or not practicing in the city requested, and 35.9% of those recommended as oculoplastic/oculofacial plastic surgeons were not oculoplastics specialists. Choice of prompt affected the result, with requests for “a doctor who does eyelid lifts” resulting in more plastic surgeons and ENTs and fewer oculoplastic surgeons. It is important to identify inaccuracies and biases in recommendations provided by AI systems as more patients may start using them to choose a surgeon.

## Introduction

Patients use a variety of methods to find surgeons, including word of mouth, referrals from other providers, lists from insurance companies, and internet searches. Previous studies have shown that patients tend to select surgeons based on factors such as surgeon reputation and competency, interpersonal skills, and affiliated hospitals [1]. More recently, artificial intelligence (AI) chatbot systems have become widely available, and patients have begun to seek medical information and self-diagnose based on chatbot responses [2]. AI chatbots are also able to recommend doctors or surgeons when prompted.

While there is great potential for AI to assist patients in aggregating data to choose a surgeon, these programs have limitations [3]. The potential for inaccuracies and bias in AI-generated responses is a significant concern. AI systems are generally trained on extensive datasets but can amplify biases present especially if there is absent, inaccurate, or misrepresented data [4]. This is especially concerning in the context of physician selection, as influence over physician recommendations can directly impact patient care and outcomes.

As patients more commonly use AI chatbot recommendations in healthcare, it is important to assess the current surgeon recommendations provided in order to uncover and address inaccuracies and biases that may be present. Furthermore, knowledge of the reasons why an AI chatbot may recommend certain providers over others can help patients decide whether those recommendations are right for them. The purpose of this study is to assess the accuracy of and biases in recommendations for oculoplastic surgeons from three commonly used chatbots.

## Materials and methods

ChatGPT version 3.5, Microsoft Bing Balanced, and Google Bard were asked for recommendations for oculoplastic surgeons practicing in the twenty cities with the highest population in the United States on April 29-30, 2023. These versions of chatbots were chosen because they were free to the public audience who may use chatbots to search for surgeons.

Each chatbot was asked the following three prompts: (1) “can you help me find an oculoplastic surgeon in (city);” (2) “can you help me find a doctor who does eyelid lifts in (city);” and (3) “can you help me find an oculofacial plastic surgeon in (city).” The same systems were also asked “can you help me find a plastic surgeon in (city).” Table 1 shows a list of the cities used. Each chatbot was prompted with the four questions for the highest populated city, followed by the four questions for the second highest populated city, and so on for all 20 cities. The chatbots returned lists of recommended surgeons in each city and all responses were recorded. 

**Table 1:** List of cities queried

CITIES
New York City
Los Angeles
Chicago
Houston
Phoenix
Philadelphia
San Antonio
San Diego
Dallas
San Jose
Austin
Jacksonville
Fort Worth
Columbus
Indianapolis
Charlotte
San Francisco
Seattle
Denver
Oklahoma City

Provider websites were identified via search engine and visited to determine the provider’s gender, specialty, and location. For oculoplastic surgeons, the American Society of Ophthalmic Plastic and Reconstructive Surgery (ASOPRS) directory was searched by name to determine whether they were an ASOPRS member. Providers who did not exist, were deceased, were not MDs, or were located in a different city were excluded. Duplicate recommendations for surgeons between the three questions or between AI systems were not excluded. Each chatbot was then asked why it made the suggestions it had by prompting with the question “why did you recommend these surgeons?”, and responses were recorded. A summary of these responses is provided in this article.

The proportions of female surgeons recommended by each chatbot and in aggregate were calculated. The national proportion of female ASOPRS members was calculated by identifying the number of female ASOPRS members in the directory on the ASOPRS website and the total number of ASOPRS members on the ASOPRS website. The proportion of female surgeons recommended by each chatbot was compared to the national proportion of female ASOPRS members using a z test.

## Results

672 total suggestions were made between the three prompts (oculoplastic surgeon; doctor who does eyelid lifts; oculofacial plastic surgeon). 19.8% of suggestions were excluded, leaving 539 suggestions.

In total, 133 suggestions made by the chatbot systems were excluded (Table 2). 38.3% of ChatGPT suggestions were excluded, 4.6% of Bing suggestions were excluded, and 15.7% of Bard suggestions were excluded. Reasons for exclusion included the provider being located in the wrong city (65.4% of exclusions), the provider name being nonexistent (25.6% of exclusions), the provider being deceased (2.3% of exclusions), the provider having retired (1.5% of exclusions), or other (social workers, physicians whose license was revoked, or recommendations for websites such as Yelp or RealSelf).

**Table 2:** Number of responses included (group and individual) versus excluded and reasons for exclusion when chatbots were prompted for surgeons in the 20 most populous cities in the United States “Other” includes social workers, physicians whose license was revoked, and recommendations for websites such as Yelp and RealSelf.

	ChatGPT		Bing		Bard		Total	
TOTAL	206		173		293		672	
EXCLUDED	79	38.3%	8	4.6%	46	15.7%	133	
Nonexistent	15	19.7%	3	75.0%	16	34.8%	34	25.6%
Wrong city	56	73.7%	1	25.0%	30	65.2%	87	65.4%
Retired	2	2.6%	0	0.0%	0	0.0%	2	1.5%
Deceased	3	3.9%	0	0.0%	0	0.0%	3	2.3%
Other	3	3.90%	4	50%	0	0.00%	7	5.3%
INCLUDED	127	61.7%	165	95.4%	247	84.3%	539	
Group	0	0.0%	72	43.6%	0	0.0%	72	13.4%
Individual	127	100.0%	91	55.2%	247	100.0%	465	86.3%

Of the 539 included suggestions, 465 were individual physicians and 72 were group practices. All 72 group practices were recommended by Bing. Out of the individual physicians recommended, 64.1% were oculoplastics specialists (Table 3). Of the remaining recommendations, 16.1% were general plastic surgery trained, 9.0% were ENT trained, 8.8% were ophthalmology but not oculoplastics trained, and 1.9% were trained in another specialty (Table 3). Other specialties included dermatology (three recommendations), pediatrics (two recommendations), internal medicine (one recommendation), interventional cardiology (one recommendation), vascular surgery (one recommendation), and hematology (one recommendation).

**Table 3:** Number of recommended surgeons who were male versus female and their subspecialty by prompt “Other” includes dermatology (3), pediatrics (2), internal medicine (1), interventional cardiology (1), vascular surgery (1) and hematology (1).

Prompt	Oculoplastic surgeon		Eyelid lifts		Oculofacial plastics		Total	
TOTAL	150		161		154		465	
GENDER								
Male	103	68.7%	122	75.8%	111	72.1%	336	72.3%
Female	47	31.3%	39	24.2%	43	27.9%	129	27.7%
SPECIALTY								
Oculoplastics	112	74.7%	75	46.6%	111	72.1%	298	64.1%
Plastic Surgery	14	9.3%	47	29.2%	14	9.1%	75	16.1%
ENT	5	3.3%	24	14.9%	13	8.4%	42	9.0%
Ophthalmology	14	9.3%	13	8.1%	14	9.1%	41	8.8%
Other	5	3.3%	2	1.2%	2	1.3%	9	1.9%

More oculoplastics specialists were suggested for certain prompts than others. When asked for an “oculoplastic surgeon” or “oculofacial plastic surgeon,” 74.7% and 72.1% of recommendations were for oculoplastics specialists, respectively. The prompt for a “doctor who does eyelid lifts,” yielded the lowest percentage (46.6%) of recommendations for oculoplastics specialists and the highest percentage of general plastic surgery trained (29.2%) and ENT trained (14.9%) physicians. Of the recommended oculoplastic surgeons, 70.1% were ASOPRS members based on the ASOPRS directory (Table 4).

**Table 4:** Number of recommended oculoplastic surgeons who were ASOPRS members ASOPRS: American Society of Ophthalmic Plastic and Reconstructive Surgery

Prompt:	Oculoplastic surgeon		Eyelid lifts		Oculofacial plastics		Total	
Oculoplastics	112		75		111		298	
ASOPRS	79	70.5%	51	68.0%	79	71.2%	209	70.1%

27.7% of recommendations across all AI systems were female physicians (Table 5). There was no statically significant difference when compared to 25.8%, the proportion of ASOPRS members in the United States who are female based on the ASOPRS member directory (p = 0.47). The proportion varied by AI system, with ChatGPT recommending 15.7% female providers (statistically significantly lower than the national proportion, p = 0.01), Bing recommending 29.7% female providers (not statistically significant, p = 0.42), and Bard recommending 33.2% female providers (statistically significantly higher than the national proportion, p = 0.02) (Table 5).

**Table 5:** Number of recommended surgeons who were male versus female and their subspecialty, by AI system *: Statistically significant difference in proportion of suggested female providers compared to the national average for ASOPRS members based on the online member directory ASOPRS: American Society of Ophthalmic Plastic and Reconstructive Surgery; AI: Artificial intelligence

	ChatGPT		Bing		Bard		Total	
TOTAL	127		91		247		465	
GENDER								
Male	107	84.3%	64	70.3%	165	66.8%	336	72.3%
Female	20	*15.7%	27	29.7%	82	*33.2%	129	27.7%
SPECIALTY								
Oculoplastics	46	36.2%	62	68.1%	190	76.9%	298	64.1%
Plastic Surgery	48	37.8%	15	16.5%	12	4.9%	75	16.1%
ENT	21	16.5%	9	9.9%	12	4.9%	42	9.0%
Ophthalmology	4	3.1%	4	4.4%	33	13.4%	41	8.8%
Other	8	6.3%	1	1.1%	0	0.0%	9	1.9%

When asked for recommendations for “plastic surgeons,” 7.3% were excluded; 90.9% of the 204 individual physicians recommended were general plastic surgery trained. The remainder were ENT (6.8%) or oculoplastics (2.3%) trained. When compared to the 73.4% of oculoplastic surgeons recommended when the systems were prompted asking for an “oculoplastic surgeon” or “oculofacial plastic surgeon” using a two proportion z score, the general plastic surgery prompt yielded significantly higher accuracy (p < 0.00001).

Each system was asked to state the reason for their individual recommendations. ChatGPT cited credentials, experience, and patient reviews. Bard cited American Board of Ophthalmology board certification, experience, credentials, and insurance acceptance. Bing stated that it used patient reviews and information on the physician websites. None of the AI systems cited ASOPRS membership as a reason for a recommendation.

## Discussion

AI has become rapidly integrated across a range of fields in healthcare, including patient care, diagnostics, and educational resources. Wearables such as smartwatches have been used to detect atrial fibrillation, and the FDA has approved fully autonomous devices for the screening of diabetic retinopathy [5-7]. Patients commonly turn to online search engines to self-educate and answer health related questions and can be expected to increasingly utilize AI chatbots [8].

Our results demonstrate high rates of inaccuracy when chatbot systems are prompted for oculoplastic surgeon recommendations. On average, nearly one out of every five suggestions made by the chatbots was excluded. When specifically prompted for an oculoplastic surgeon or an oculofacial plastic surgeon, over 25% of recommendations were not oculoplastics trained surgeons. This level of inaccuracy poses a challenge for any patient seeking a provider recommendation via a chatbot. This high rate of nonexistent answers has previously been documented with chatbots and called artificial hallucinations; newer versions of chatbots may have lower rates of hallucinations but they have not been eliminated [9].

Prior research has similarly questioned the accuracy of AI chatbot recommendations in healthcare. Studies have shown mixed results when investigating whether AI chatbots can accurately answer questions patients may have about their health. One study showed that ChatGPT could provide largely appropriate responses when asked about gastroesophageal reflux disease, while another showed that ChatGPT gave output related to neurosurgical conditions of only “fair” quality that was significantly inferior to the American Association of Neurological Surgeons website [10,11]. Yet another study showed that chatbots provided comparable quality to information about hepato-pancreato-biliary information available on the Internet [12].

The accuracy of these chatbots may vary by subspecialty and topic. For common retinal diseases, ChatGPT was found to give consistently appropriate answers for most questions, but the answers were difficult to read [13]. For lacrimal drainage disorders, ChatGPT was found to have high error rates, giving accurate responses to only 40% of questions [14]. In our study, in comparison to the high rates of inaccuracy for oculoplastic surgeon recommendations, when the systems were prompted with “can you help me find a plastic surgeon in (city)” over 90% of physicians recommended were general plastic surgeons. Subspecialties with fewer surgeons present in each city may be prone to more bias than those with higher numbers.

These results also show the importance of the words used when prompting AI systems. When asking AI chatbots the same question, a different phrasing can lead to a different response. Here, more oculoplastic surgeons were recommended when the chatbots were specifically asked for an oculoplastic surgeon or an oculofacial plastic surgeon than when they were asked for a doctor who does eyelid lifts. The prompts that patients enter when searching for a doctor may vary significantly and may be impacted by other factors such as region or familiarity with medical terminology.

In this study, we chose prompts that included the words “doctor” or “surgeon.” However, if patients use prompts looking for a solution to their drooping eyelids or other medical issues that exclude these words, it is possible that providers who are not trained as surgeons may be recommended by these AI systems. Future research can help elucidate when and why these systems may recommend certain types of providers over others.

In our study, bias in gender of provider recommended varied greatly by AI system, with two of the three chatbots having a statistically significant difference in proportion of female providers recommended compared with the nationwide proportion of female ASOPRS members. While the proportion of female ASOPRS members may not be directly representative of the proportion of female oculoplastic surgeons, it was used as a proxy metric. It is known that algorithmic bias in AI systems can replicate real world social biases [15]. ASOPRS membership has changed over time, with a greater number of women entering the field of oculofacial plastic surgery; chatbot answers, which are pulled from historic data, may similarly change. Philosophical and ethical concerns have been raised about the use of new AI technologies that may be trained using inherently biased datasets, and may influence existing inequities if advancements are not distributed equally among groups [16,17]. Similarly, these differences in AI models may lead some users to receive results with more inherent bias than other models. It can be difficult to obtain transparency regarding why an AI chatbot is giving a particular answer, but this information is critical in reducing inherent biases.

For the three AI chatbots tested in this study, reasons for suggesting a physician varied. Board certification in ophthalmology was cited as a reason to recommend a surgeon, but ASOPRS membership was not cited as a reason for any of the AI systems. Certain AI systems used information on physician websites while others used patient reviews. Since surgeons have control over the information that appears on their websites, knowing what pieces of information the AI systems are searching for can help in patient recruitment. This information can also help train future AI models to make less biased recommendations.

Limitations in our study include the use of a limited number of prompts. With different prompts, each AI system may have yielded different levels of accuracy or bias. Further investigations will be important to assess how best to elicit accurate responses when asking for provider recommendations. Additionally, the search was conducted at one static time point, while AI engines update themselves frequently. Finally, there may be sources of bias besides gender that were not collected as variables and that we were therefore not able to uncover in this study. Future studies are needed to identify and assess additional sources of bias.

## Conclusions

As AI rapidly progresses, patients are more likely to use chatbot systems to seek answers in healthcare. In particular, patients may use chatbots to request recommendations for healthcare providers. This study shows that there are high rates of inaccuracy when using chatbots for surgeon recommendations, with many of the recommended surgeons being nonexistent, not practicing in the city requested, or not trained in the specialty requested. The phrasing of each prompt also impacts surgeon recommendations and word choice may be influenced by other patient factors such as medical literacy. As patients use chatbots more frequently, it is important to continually assess whether the responses these systems provide are accurate and identify any inherent biases.

## References

[1] Yahanda AT, Lafaro KJ, Spolverato G, Pawlik TM (2016). A systematic review of the factors that patients use to choose their surgeon. World J Surg.

[2] Shahsavar Y, Choudhury A (2023). User intentions to use ChatGPT for self-diagnosis and health-related purposes: cross-sectional survey study. JMIR Hum Factors.

[3] Goyal D, Guttag J, Syed Z, Mehta R, Elahi Z, Saeed M (2020). Comparing precision machine learning with consumer, quality, and volume metrics for ranking orthopedic surgery hospitals: retrospective study. J Med Internet Res.

[4] Norori N, Hu Q, Aellen FM, Faraci FD, Tzovara A (2021). Addressing bias in big data and AI for health care: a call for open science. Patterns (N Y).

[5] Bedi A, Al Masri MK, Al Hennawi H, Qadir S, Ottman P (2023). The integration of artificial intelligence into patient care: a case of atrial fibrillation caught by a smartwatch. Cureus.

[6] Lim JI, Regillo CD, Sadda SR, Ipp E, Bhaskaranand M, Ramachandra C, Solanki K (2023). Artificial intelligence detection of diabetic retinopathy: subgroup comparison of the EyeArt System with ophthalmologists' dilated examinations. Ophthalmol Sci.

[7] Dow ER, Khan NC, Chen KM (2023). AI-human hybrid workflow enhances teleophthalmology for the detection of diabetic retinopathy. Ophthalmol Sci.

[8] Tan SS, Goonawardene N (2017). Internet health information seeking and the patient-physician relationship: a systematic review. J Med Internet Res.

[9] Alkaissi H, McFarlane SI (2023). Artificial hallucinations in ChatGPT: implications in scientific writing. Cureus.

[10] Henson JB, Glissen Brown JR, Lee JP, Patel A, Leiman DA (2023). Evaluation of the potential utility of an artificial intelligence chatbot in gastroesophageal reflux disease management. Am J Gastroenterol.

[11] Mishra A, Begley SL, Chen A, Rob M, Pelcher I, Ward M, Schulder M (2023). Exploring the intersection of artificial intelligence and neurosurgery: let us be cautious with ChatGPT. Neurosurgery.

[12] Walker HL, Ghani S, Kuemmerli C, Nebiker CA, Müller BP, Raptis DA, Staubli SM (2023). Reliability of medical information provided by ChatGPT: assessment against clinical guidelines and patient information quality instrument. J Med Internet Res.

[13] Momenaei B, Wakabayashi T, Shahlaee A (2023). Appropriateness and readability of ChatGPT-4-generated responses for surgical treatment of retinal diseases. Ophthalmol Retina.

[14] Ali MJ (2023). ChatGPT and lacrimal drainage disorders: performance and scope of improvement. Ophthalmic Plast Reconstr Surg.

[15] Saint James Aquino Y (2023). Making decisions: bias in artificial intelligence and data‑driven diagnostic tools. Aust J Gen Pract.

[16] Fisher E, Flynn MA, Pratap P, Vietas JA (2023). Occupational safety and health equity impacts of artificial intelligence: a scoping review. Int J Environ Res Public Health.

[17] Tang L, Li J, Fantus S (2023). Medical artificial intelligence ethics: a systematic review of empirical studies. Digit Health.

